# Care Technician Staffing Ratios in Dialysis Units: In Search of a Balance Between Workforce and Patient Safety

**DOI:** 10.1016/j.xkme.2024.100795

**Published:** 2024-02-15

**Authors:** Hanna Webb, Jay B. Wish

**Affiliations:** Division of Nephrology, Indiana University School of Medicine, Indianapolis, IN

Related article, p ●

Hemodialysis (HD) staff to patient ratios have been a topic of debate since the inception of maintenance dialysis therapy, with no clear consensus on whether regulation should mandate minimum ratios in outpatient facilities. This issue recently garnered attention in California with proposition SB 349, which sought to mandate outpatient dialysis staffing ratios of 1 dialysis nurse per 12 patients and 1 dialysis patient care technician (PCT) for 4 patients. The measure was ultimately withdrawn because of lack of support from the governor and concerns about the already critical nursing shortage in California and nationwide. Research by Plantinga et al^1^ in this issue of *Kidney Medicine* helps provide context to the debate by analyzing patient to PCT staffing ratios over time throughout the country. This investigation of recent and historical trends prompts more questions to be considered and raises issues for future research.

PCTs represent a large portion of the outpatient dialysis workforce. The Centers for Medicare and Medicaid Services (CMS) 2022 Dialysis Facility Report quantifies over 100,000 dialysis facility employees nationwide. In total, 45% of these employees were PCTs, and 90% of PCTs worked full time in their role.^2^ PCTs are expected to perform most of the tasks associated with a dialysis treatment, including setting up the machine; accessing a venous catheter, fistula or graft; monitoring the patient; documenting the therapy; and turning over each station between treatments. For most patients, PCTs are the main caretaker at dialysis. They are required by CMS Conditions for Coverage to undergo on-the-job training and take a certification examination to ensure competency before practice with less direct supervision.^3^

The CMS Conditions for Coverage notably do not specify minimum staffing ratios, with the CMS instead focusing on quality measures that are publicly reported and affect reimbursement to hold dialysis facilities accountable for the care provided. This assumes each facility will keep staffing ratios at a level needed for the best possible performance. Eight states (Georgia, Maryland, Massachusetts, New Jersey, Oregon, South Carolina, Texas, and Utah) and the District of Columbia do have state-mandated minimum staffing ratios for outpatient dialysis facilities.

The report by Plantinga et al^1^ is an ecological study of PCT staffing patterns in United States HD facilities, examining point prevalent data from 2019 in detail and trending data from 2004 to 2019 for both staffing ratios and percentage of open PCT positions. The major data sources are the Annual Facility Surveys, Dialysis Patient Compare, Medical Evidence Report (Form 2728 completed at dialysis initiation), and United States Renal Data System. The authors obtained data from an impressive 6,862 facilities for the year 2019 and then calculated the ratios based on HD patient census at the facility and number of PCTs. The average patient to PCT ratio was 9.9, although there was significant variability at the state and regional level. Midwestern and Southern regions as well as New York and New Jersey had the highest ratios. The Pacific Northwest had the lowest ratios. The lowest quartile of centers had patient to PCT ratios less than 8.2, whereas the highest quartile had ratios greater than 12. There was a decrease in the national ratio from 10.6 to 9.9 between 2004 and 2019, although the percent of open PCT positions increased from 2.8% to 3.5%.

Facilities with higher patient to PCT ratios were generally larger facilities associated with a large dialysis organization. Higher patient to PCT ratios were linked to facility characteristics, including location in higher poverty areas, higher percentage of patients with functional impairment, less permanent dialysis access, and lower numbers of transplant listings. It should be noted that the authors of this study were unable to account for the acuity of patients and did not have information regarding the number of shifts PCTs worked. Despite these limitations, the results of this study are consistent with a 2013 study from Yoder et al^4^ that reported average staffing ratios nationwide that were highest in large units. In this 2013 study, the lowest patient to PCT ratios were found in nonchain freestanding facilities.

The importance of this topic cannot be overstated and is becoming more urgent because of the lack of evidence-based solutions to date and the potential effect on patient and provider safety and wellness. It is known that higher patient to nurse ratios are associated with more tasks left undone and more adverse events, including hypotension and skipped or shortened dialysis treatments.^5^ Fewer staff per patient ratios have also been correlated with more 30-day readmissions and hepatitis C virus de novo infections.^6^, ^7^, ^8^ Infections and readmissions are metrics in the End-Stage Renal Disease Quality Incentive Program (QIP) and can affect facility reimbursement through CMS. The emphasis on infection control in the QIP is at least in part due to a history of poor oversight regarding basic infection prevention measures.^9^ When accounting for dialysis-related infections, it should be considered that PCT roles include station turnover between patients, changing gloves and protective equipment, exit site care of vascular access, and station sanitation. All these activities take time, especially when changing over multiple stations during a short period, and PCTs caring for more patients have less time per turnover. Notably, recent California legislation has also sought to limit the amount of time per turnover.^10^

There is some evidence that staffing ratios have no direct effect on dialysis patient outcomes, suggesting a more complex relationship that involves other unaccounted for factors suitable for future research.^11^ The 8 states and District of Columbia with mandated staffing ratios do not boast improved quality measures in the End-Stage Renal Disease QIP data and have not shown improved hospitalizations or mortality rates.^10^^,^^12^ Although mandated staffing ratios are much lower than the average throughout the country (4 patients to 1 PCT vs 10 patients to 1 PCT), most states with staffing ratio mandates did not come close to meeting these ratios in the study by Plantinga et al,^1^ a finding that is difficult to explain.

There are important policy implications of this uncertainty. First, the conflicting data on how staffing ratios affect quality of care make it challenging to suggest an evidence-based staffing ratio that could be mandated on a national level. Second, it is not clear that this would be the correct approach, as many experts have suggested incorporating patient acuity into staffing ratio mandates for a more individualized approach. In 1998, Freund^13^ developed an acuity model for patients receiving maintenance HD, estimating the caregiver time requirement for each individual. An acuity-based staffing regulation was also considered in the comments of the 2008 CMS Conditions for Coverage. This was rejected given the limited evidence to support it and multitude of other facility-level factors to be considered when determining appropriate staffing, including staff skill level and experience, efficiency of systems, and varying documentation demands.

The report by Plantinga et al^1^ is particularly concerning because facilities caring for the most economically disadvantaged patients have the highest patient to staff ratios. This is an issue that requires further investigation as the medical field continues to grapple with the myriad of ways socioeconomic factors affect patient outcomes. Potential contributing factors include a less advantageous payer mix in higher poverty areas limiting staffing budgets and a lack of eligible personnel in a certain location because PCTs are required by the CMS to have a high school diploma and commit to a training and certification program over at least 6 months.^3^ The finding of higher patient to nurse ratios in facilities with higher patient to PCT ratios indicates a global staffing issue at some facilities as opposed to the use of PCTs as a low-cost replacement for nurses, as has been suggested in other research.^4^

The lack of regulatory guidance and evidence regarding safe staffing ratios leaves many PCTs dependent on their employer to determine an appropriate daily workload. This can be quite subjective, as supported by the high variability of staffing ratios at facilities across the country.^1^ Uncertainty around staffing ratios will increasingly become problematic as health care and dialysis providers attempt to rebound from the coronavirus disease 2019 pandemic, which likely increased the patient to PCT ratios reported in the study by Plantinga et al,^1^ and stretched dialysis facility staffing budgets even further. At the same time, the number of patients with kidney failure requiring dialysis continues to increase yearly, and PCT turnover and burnout levels remain high.^14^

Regardless of the conflicting evidence base for a prescribed patient to PCT staffing ratio, this issue has to be prioritized in both policy considerations and individual dialysis facilities to promote PCT retention and wellbeing as well as patient safety. PCTs are struggling with a lack of empowerment and purpose in their work, despite acting as the main caregiver for patients receiving dialysis. PCT attrition and dissatisfaction will surely worsen with unregulated workloads and high-stress work environments, further exacerbating the critical workforce shortage being faced in nephrology.
